# Combined treatment of mitoxantrone sensitizes breast cancer cells to rapalogs through blocking eEF-2K-mediated activation of Akt and autophagy

**DOI:** 10.1038/s41419-020-03153-x

**Published:** 2020-11-03

**Authors:** Yidi Guan, Shilong Jiang, Wenling Ye, Xingcong Ren, Xinluan Wang, Yi Zhang, Mingzhu Yin, Kuansong Wang, Yongguang Tao, JinMing Yang, Dongsheng Cao, Yan Cheng

**Affiliations:** 1 Department of Pharmacy, The Second Xiangya Hospital, Central South University, 410011 Changsha, Hunan China; 2 Xiangya School of Pharmaceutical Sciences, Central South University, 410008 Changsha, Hunan China; 3 Department of Cancer Biology and Toxicology, Department of Pharmacology, College of Medicine, Markey Cancer Center, University of Kentucky, Lexington, KY 40536 USA; 4 Translational Medicine R&D Center, Institute of Biomedical and Health Engineering, Shenzhen Institutes of Advanced Technology, Chinese Academy of Sciences, 518057 Shenzhen, China; 5 Department of Pharmacology, College of Pharmaceutical Sciences, Soochow University, Suzhou, China; 6 Department of Dermatology, Hunan Engineering Research Center of Skin Health and Disease, Hunan Key Laboratory of Skin Cancer and Psoriasis, Xiangya Hospital, Central South University, 410008 Changsha, Hunan China; 7 Department of Pathology, Xiangya Hospital, Central South University, 410078 Changsha, China; 8 Cancer Research Institute, School of Basic Medicine, and Key Laboratory of Carcinogenesis and Cancer Invasion, Ministry of Education, Central South University, 410008 Changsha, Hunan China

**Keywords:** Breast cancer, Cell signalling

## Abstract

Oncogenic activation of the mTOR signaling pathway occurs frequently in tumor cells and contributes to the devastating features of cancer, including breast cancer. mTOR inhibitors rapalogs are promising anticancer agents in clinical trials; however, rapalogs resistance remains an unresolved clinical challenge. Therefore, understanding the mechanisms by which cells become resistant to rapalogs may guide the development of successful mTOR-targeted cancer therapy. In this study, we found that eEF-2K, which is overexpressed in cancer cells and is required for survival of stressed cells, was involved in the negative-feedback activation of Akt and cytoprotective autophagy induction in breast cancer cells in response to mTOR inhibitors. Therefore, disruption of eEF-2K simultaneously abrogates the two critical resistance signaling pathways, sensitizing breast cancer cells to rapalogs. Importantly, we identified mitoxantrone, an admitted anticancer drug for a wide range of tumors, as a potential inhibitor of eEF-2K via a structure-based virtual screening strategy. We further demonstrated that mitoxantrone binds to eEF-2K and inhibits its activity, and the combination treatment of mitoxantrone and mTOR inhibitor resulted in significant synergistic cytotoxicity in breast cancer. In conclusion, we report that eEF-2K contributes to the activation of resistance signaling pathways of mTOR inhibitor, suggesting a novel strategy to enhance mTOR-targeted cancer therapy through combining mitoxantrone, an eEF-2K inhibitor.

## Introduction

Mammalian target of rapamycin (mTOR) is a serine/threonine protein kinase in the PI3K-related kinase (PIKK) family. mTOR functions through two well-described multiprotein complexes: mTOR complex 1 (mTORC1) and mTOR complex 2 (mTORC2)^1^. mTORC1 is a central controller that integrates nutrient, growth factor/hormone, and stress signaling to regulate cellular metabolism, mRNA translation, and cell growth/proliferation^2^. The best-characterized function of mTORC2 comes from its regulation of Akt through the direct phosphorylation of Akt at S473^3^. Deregulation of the mTOR signaling pathway is one of the most commonly observed pathological alterations in human cancers. mTORC1 stays downstream of oncogenic pathways, such as PI3K/Akt and Ras/Raf/Mek/ERK^4^. Mutations of the oncogenic pathway or inactivation of tumor suppressors such as PTEN and p53 all promote overactivation of mTORC1. Carcinogenic activation of mTOR is involved in the process required for tumor cell survival, growth, and proliferation^5^.

Direct pharmacologic inhibition of mTOR signaling is therefore an attractive therapeutic strategy for cancer treatment. Rapamycin, an established mTOR inhibitor, is effective as an immunosuppressant and anti-proliferation agent^6,7^. Rapamycin and its analogues, such as temsirolimus and everolimus, have been approved by the Food and Drug Administration (FDA) to treat advanced renal cell carcinoma and metastatic breast cancer^8,9^. Despite the promising anticancer activity of mTOR inhibitors observed in preclinical and clinical studies, the overall outcomes of rapalogs as a monotherapy are disappointed. There are several explanations for this limited efficacy. The first comes from the induction of Akt activation through the mTORC1/S6K-dependent negative-feedback loops; thus Akt is tightly associated with the development of cell resistance to rapalogs^10,11^. Accordingly, mTORC1/mTORC2 dual inhibitors and co-targeting PI3K/Akt signaling were derived to overcome rapalogs resistance caused by activation of negative-feedback carcinogenic pathways^12^. Second, the suboptimal outcomes obtained from the activation of autophagy induced by rapalogs^13^. Autophagy is an evolutionarily conserved process through which intracellular components are degraded for recycling use. For tumor tissues, autophagy is hijacked by cancer cells to meet the increased energy requirements, which are essential for tumor survival and progression^14^. To solve this lack of virtue, combining rapalogs with autophagy inhibitors are highly recommended. A phase I clinical trial reported that a combination of temsirolimus and autophagy inhibitor hydroxychloroquine showed significant anticancer activity in patients with melanoma^15^. Therefore, an in-depth study of acquired resistances of rapalogs at the molecular level will help enhance the anticancer effect of this kind of drug and will be beneficial to their future clinical application.

Eukaryotic elongation factor-2 kinase (eEF-2K), a calcium/calmodulin-dependent kinase, inactivates the only known substrate eEF2 by phosphorylating at Thr56, thereby hindering its ability to bind ribosomes and inhibiting the elongation step of protein synthesis^16^. Tumor cells grow in an acidic, anoxic microenvironment with poor blood supply, anabolic processes driven by carcinogenic pathways can consume vast amounts of energy, which will cause fatal damage to cancer cells. Thus, cancer cells evolve a new set of signal transduction system to adapt to the metabolic pressures. As a vital part, eEF-2K is activated in response to various environmental or metabolic stresses to promote tumor cell survival^17^. Of particular importance is its role in regulating autophagy, a growing data including our own support the notion that eEF-2K-mediated autophagy aids the growth of cancer cells in vitro and in vivo in response to metabolic or therapeutic stresses^18,19^. In addition, our study demonstrated that eEF-2K also facilitates tumor cells growth by promoting a shift from oxidative phosphorylation to glycolysis, and inhibiting eEF-2K can switch off the metabolic process tumor cells rely on and increase the sensitivity of tumor cells to chemotherapy^20^. Therefore, eEF-2K is regarded as a valid target for anticancer therapy.

In this study, we demonstrated that eEF-2K is an important mediator in the activation of Akt and autophagy induced by rapalogs, and inhibition of eEF-2K can turn off both cytoprotective pathways and significantly enhanced the anti-proliferation effect of rapalogs in breast cancer cells. Importantly, we identified mitoxantrone as an eEF-2K inhibitor based on docking-based virtual screening, SPR binding, and activity analysis. Combining rapalogs with mitoxantrone can disrupt the resistance mechanism of mTOR inhibitors and exert a highly synergistic antitumor effect in vitro and in vivo.

## Results

### An eEF-2K-eEF2-AMPK signaling pathway is involved in the Akt activation induced by mTOR inhibitor

**Fig. 1:**
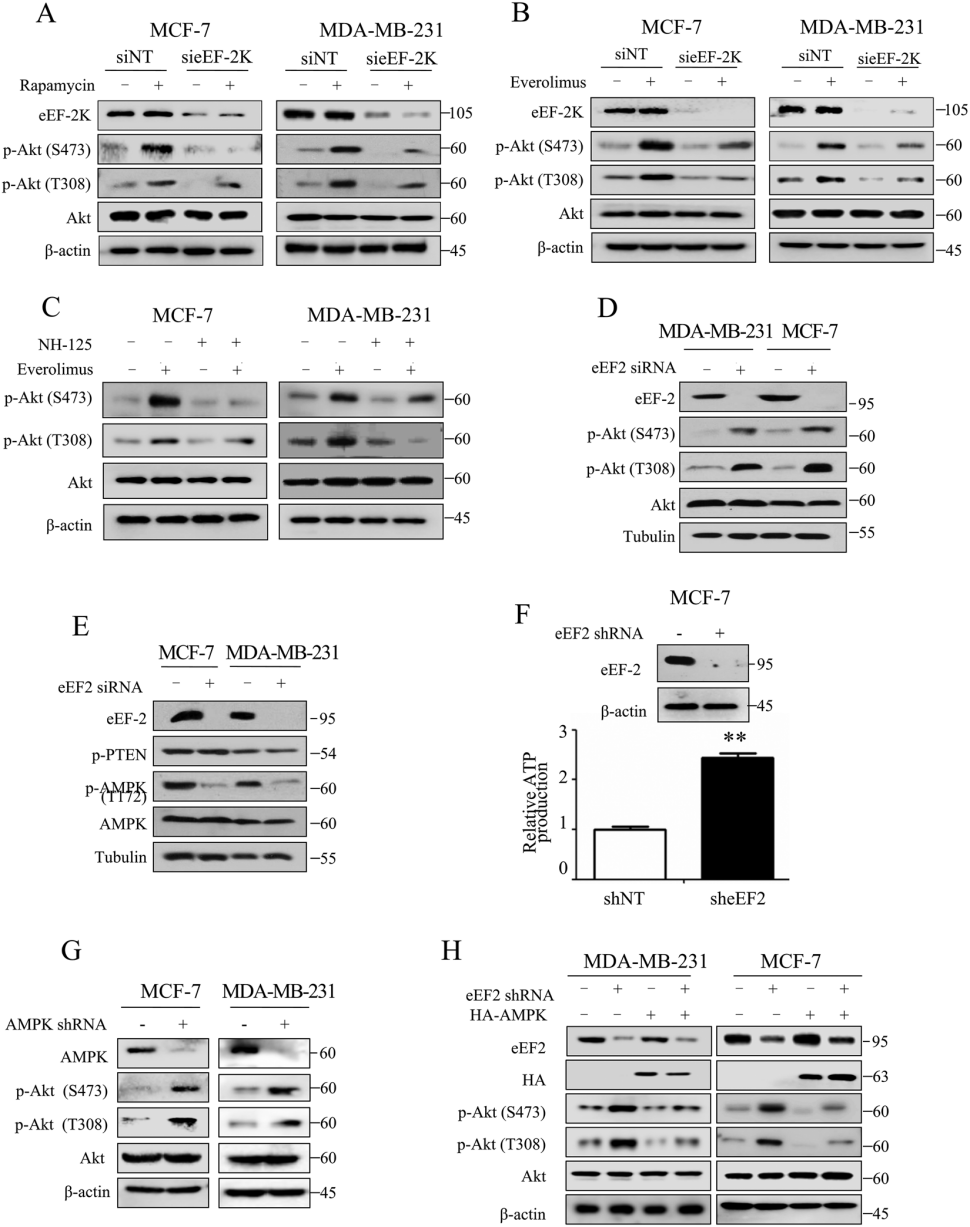
eEF-2K-eEF2-AMPK signaling pathway is involved in the Akt activation induced by mTOR inhibitor. MDA-MB-231 or MCF-7 cells were transfected with nontargeting siRNAs or siRNAs targeting eEF-2K, followed by treatment with 100 nM rapamycin (**A**) or everolimus (**B**) for 24 h. Cell lysates were analyzed by western blot for levels of indicated proteins. β-actin was used as a loading control. **C** MDA-MB-231 or MCF-7 cells were pretreated with 0.25 μM NH125 for 1 h, followed by treatment with 100 nM everolimus for 24 h. Cell lysates were analyzed by western blot for levels of indicated proteins. β-actin was used as a loading control. **D**, **E** MDA-MB-231 or MCF-7 cells were transfected with nontargeting siRNAs or siRNAs targeting eEF2. Cell lysates were analyzed by western blot for levels of indicated proteins. β-actin was used as a loading control. **F** The content of ATP in MCF-7 cells (shCtrl and sheEF2) was analyzed by the ATPlite Luminescence Assay Kit. The results are displayed as the means ± SD of triplicate measurements from one of three identical experiments; **P < 0.01, *t* test. **G** MCF-7 and MDA-MB-231 cells were transfected with nontargeting shRNA or shRNA targeting AMPK. Cell lysates were analyzed by western blot for levels of indicated proteins. β-actin was used as a loading control. **H** MCF-7 and MDA-MB-231 cells (shCtrl and sheEF2) were further transfected with HA-AMPK. Cell lysates were analyzed by western blot for levels of indicated proteins. β-actin was used as a loading control. All experiments were repeated three times. *P < 0.05, **P < 0.01, and ***P < 0.001.

As shown in Supplementary Fig. 1, the exposure of breast cancer cells to rapamycin not only suppressed the activity of the mTORC1 axis molecules such as decreased p-p70S6K (T389) and p-S6 (S240/244) but also caused a marked increase in p-Akt both at S473 and T308 in a time- and dose-dependent manner. Meanwhile, we found that rapamycin induced an increase in p-eEF2, indicating the activation of eEF-2K (Supplementary Fig. 1), which is in line with previous studies that mTOR and its downstream p70S6K are negative regulators of eEF-2K^21,22^. We next measured the association between eEF-2K and the negative-feedback activation of Akt induced by rapalogs. Suppression of eEF-2K by RNA interference dramatically decreased the phosphorylation of Akt induced by rapamycin and everolimus (Fig. 1A, B). In addition, NH125, a commercial eEF-2K inhibitor, also suppressed the activation of Akt in everolimus-treated breast cancer cells (Fig. 1C). Collectively, these results indicate that eEF-2K mediates rapalogs-induced Akt activation.

To explore the molecular mechanism linking Akt activation and eEF-2K, we tested the role of eEF2, the only known substrate of eEF-2K. As shown in Fig. 1D, silencing eEF2 in both MDA-MB-231 and MCF-7 cells caused a remarkable elevation in the levels of p-Akt. To avoid off-target effects of siRNA, we used another two different sequences of eEF2-siRNA and got the same result that eEF2 silencing significantly upregulated the expression of p-Akt (Supplementary Fig. 2).

To investigate how eEF2 regulates the activity of Akt, we assessed the effect of eEF2 on activities of AMPK and PTEN, both upstream regulators of Akt. Figure 1E shows that there was no significant change in the level of p-PTEN, but the expression level of p-AMPK (T172) was markedly decreased in eEF2 knockdown cells, indicating that eEF2 may regulate Akt activity through AMPK. AMPK is a well-known intracellular energy sensor directly regulated by the ratio of AMP/ATP. As protein synthesis is a major energy-consuming process, knocking down eEF2 may lead to the accumulation of energy by impairing the process of peptide chain elongation. We observed an increased amount of ATP in the eEF2 knockdown cells (Fig. 1F), indicating that suppression of eEF2 contributes to energy saving, thus restraining AMPK activity.

Consistent with previous reports that activated AMPK stimulates the dephosphorylation of Akt^23^, we found that silencing AMPK expression indeed resulted in an increase in Akt activity, suggesting that AMPK inhibits the activation of Akt in breast cancer cells (Fig. 1G). To validate eEF2 does regulate Akt activity through AMPK, we transfected an AMPK expression plasmid in the eEF2 knockdown cells. Notably, upregulation of p-Akt at S473 and T308 in the eEF2 knockdown cells was turned down by ectopic expression of AMPK (Fig. 1H). These results provide evidence that eEF-2K regulates Akt activity through eEF2-AMPK.

### Inhibition of eEF-2K abrogates rapalogs-induced autophagy

**Fig. 2:**
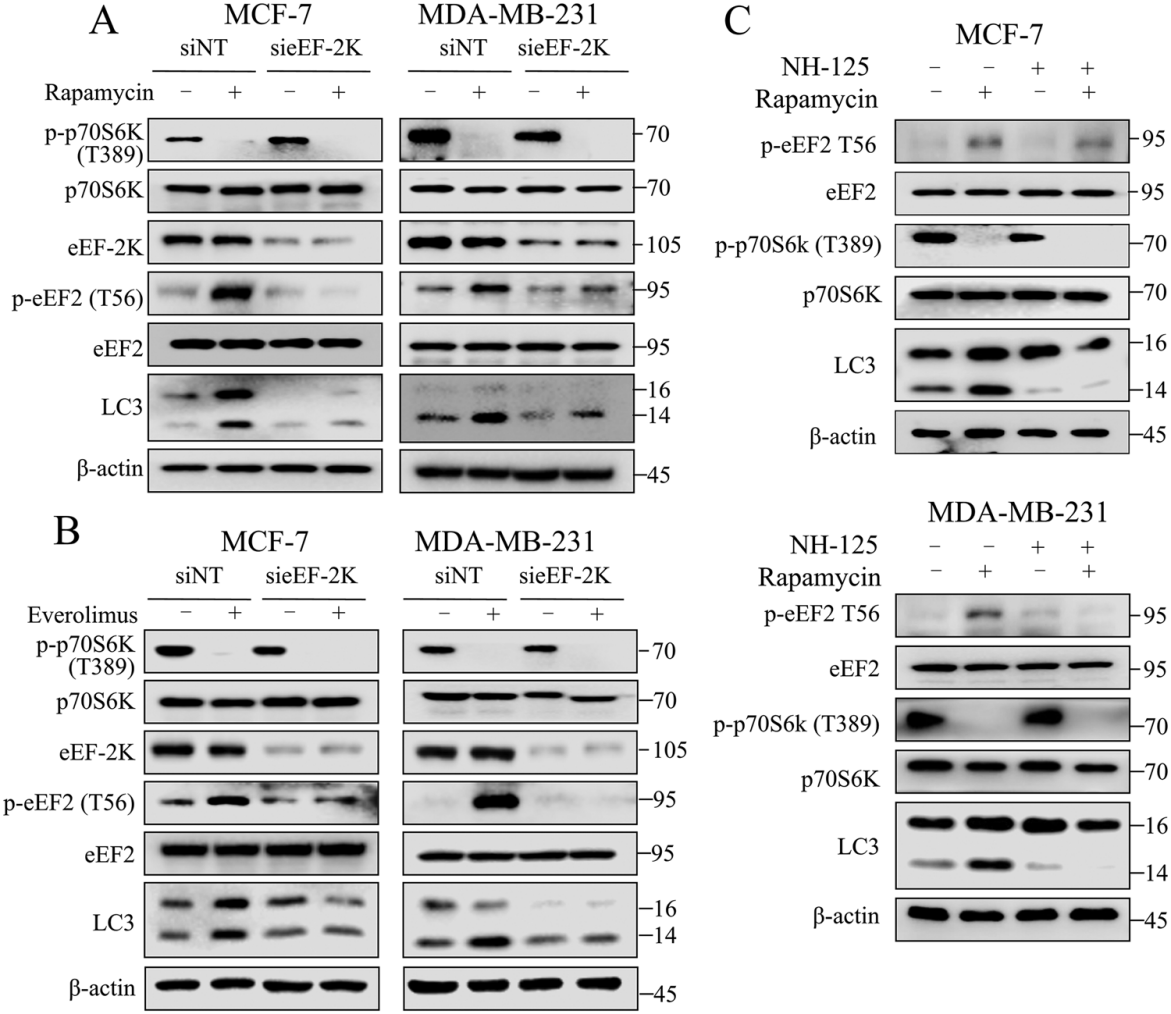
Inhibition of eEF-2K abrogates rapalogs-induced autophagy. MDA-MB-231 or MCF-7 cells were transfected with nontargeting siRNAs or siRNAs targeting eEF-2K, followed by treatment with 100 nM rapamycin (**A**) or everolimus (**B**) for 24 h. Cell lysates were analyzed by western blot for levels of indicated proteins. β-actin was used as a loading control. **C** MDA-MB-231 or MCF-7 cells were pretreated with 0.25 μM NH125 for 1 h, followed by treatment with 100 nM rapamycin for 24 h. Cell lysates were analyzed by western blot for levels of indicated proteins. β-actin was used as a loading control. All experiments were repeated three times.

In mammalian cells, mTORC1 inhibits the autophagy-initiating complex by phosphorylating autophagy-related gene 13 (ATG13) and ULK1/2^24^. Therefore, inhibition of mTORC1 leads to autophagy induction, helping maintain cancer cell survival. When tumor cells are exposed to nutrient deprivation or therapeutic stress, eEF-2K plays an important role in promoting tumor survival and causing chemotherapy resistance by activating autophagy. Here, we sought to explore whether eEF-2K has involved in mTOR inhibitors-induced autophagy. As shown in Fig. 2A, when cancer cells are exposed to rapamycin, there is a robust elevation of p-eEF2 at Thr 56, indicating the activation of eEF-2K. Depletion of eEF-2K attenuated rapamycin-induced autophagy, as determined by a decrease in autophagy hallmark LC3 II. Similarly, inhibition of eEF-2K also blocked everolimus-induced autophagy (Fig. 2B). In addition, inactivating eEF-2K using NH125 also suppressed the autophagic effect triggered by rapamycin (Fig. 2C). These results clearly suggest that eEF-2K mediates mTORC1 inhibition-induced autophagy.

### Inhibition of eEF-2K enhanced the anticancer effect of mTOR inhibitors in vitro and in vivo

**Fig. 3:**
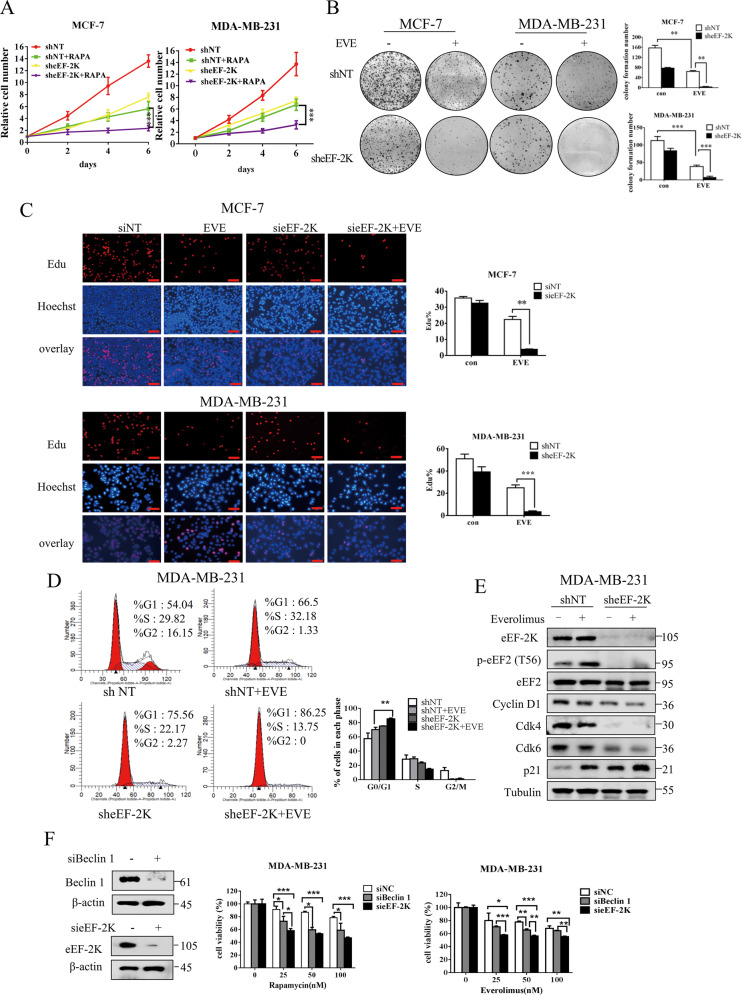
Inhibition of eEF-2K enhanced the anticancer effect of mTOR inhibitors in vitro. **A** MCF-7 or MDA-MB-231 cells (shCtrl and sheEF-2K) were treated with 100 nM rapamycin for indicated durations. Cell proliferation was analyzed by cell counting. **P < 0.01, ***P < 0.001, *t* test. **B** MCF-7 or MDA-MB-231 cells (shCtrl and sheEF-2K) were seeded for colony-formation assay. The results are displayed as means ± SD (*n* = 3). **P < 0.01, ***P < 0.001, *t* test. **C** MCF-7 cells which were transfected with nontargeting siRNAs or siRNAs targeting eEF-2K or MDA-MB-231 cells (shCtrl and sheEF-2K) followed by treatment with 100 nM everolimus for 24 h. Cell proliferation was detected by Edu incorporation assay. The results are displayed as means ± SD (*n* = 3). **P < 0.01, ***P < 0.001, *t* test. Scale bar: 100 μm. **D** MDA-MB-231 cells (shCtrl and sheEF-2K) were treated with 100 nM everolimus for 48 h. Cell cycle was assessed by PI staining and flow cytometry. **E** MDA-MB-231 cells (shCtrl and sheEF-2K) were treated with 100 nM everolimus for 48 h. Cell cycle-related proteins were analyzed by western blot. Tubulin was used as a loading control. **F** MDA-MB-231 cells were transfected with nontargeting siRNAs or siRNAs targeting eEF-2K or siRNAs targeting Beclin 1, followed by treatment with different concentrations of rapamycin or everolimus for 72 h. Cell viability was analyzed by CCK8 reagent. The results are displayed as the means ± SD of triplicate measurements from one of three identical experiments; *P < 0.05, **P < 0.01, ***P < 0.001, *t* test.

We next wanted to know whether eEF-2K-mediated activation of pro-survival signaling pathways affects the anticancer effects of rapalogs. As shown in Fig. 3A, the proliferation of cells treated with eEF-2K knockdown and rapamycin was significantly reduced compared to cells treated with rapamycin alone. Furthermore, breast cancer cells with eEF-2K knockdown and rapamycin formed fewer colonies, as compared with cells treated with everolimus alone (Fig. 3B). Edu incorporation assay was further performed to measure cell proliferation. Figure 3C shows that in the everolimus-treated cells, eEF-2K deletion caused a marked reduction in the percentage of Edu-positive cells, indicating that eEF-2K facilitates the entry of cells into the S phase. In agreement with Edu results, silencing eEF-2K caused an accumulation of cells in the G_0_/G_1_ population and a reduction in S and G_2_/M population in everolimus-treated breast cancer cells (Fig. 3D), indicating that suppression of eEF-2K further caused G_0_/G_1_ cell cycle arrest induced by mTOR inhibitor. Additionally, cyclin D1 and Cdk 4, Cdk 6 (the catalytic subunits of cyclin D1), which facilitate the transition of the cell cycle from G_0_/G_1_ phase into the S phase^25^, exhibited a marked reduction. By contrast, Cdk inhibitor p21 was increased in the cells combination treatment of everolimus and eEF-2K knockdown compared to everolimus alone treatment (Fig. 3E). To further demonstrate the effect of eEF-2K suppression on the sensitivity of mTOR inhibitor against breast cancer cell, we compared the cell viability in response to combined treatment of mTOR inhibitor with eEF-2K siRNA or Beclin 1 siRNA. Figure 3F shows that compared with inhibiting autophagy by silencing beclin 1, inhibition of eEF-2K dramatically rendered the cancer cells more sensitive to rapalogs.

**Fig. 4:**
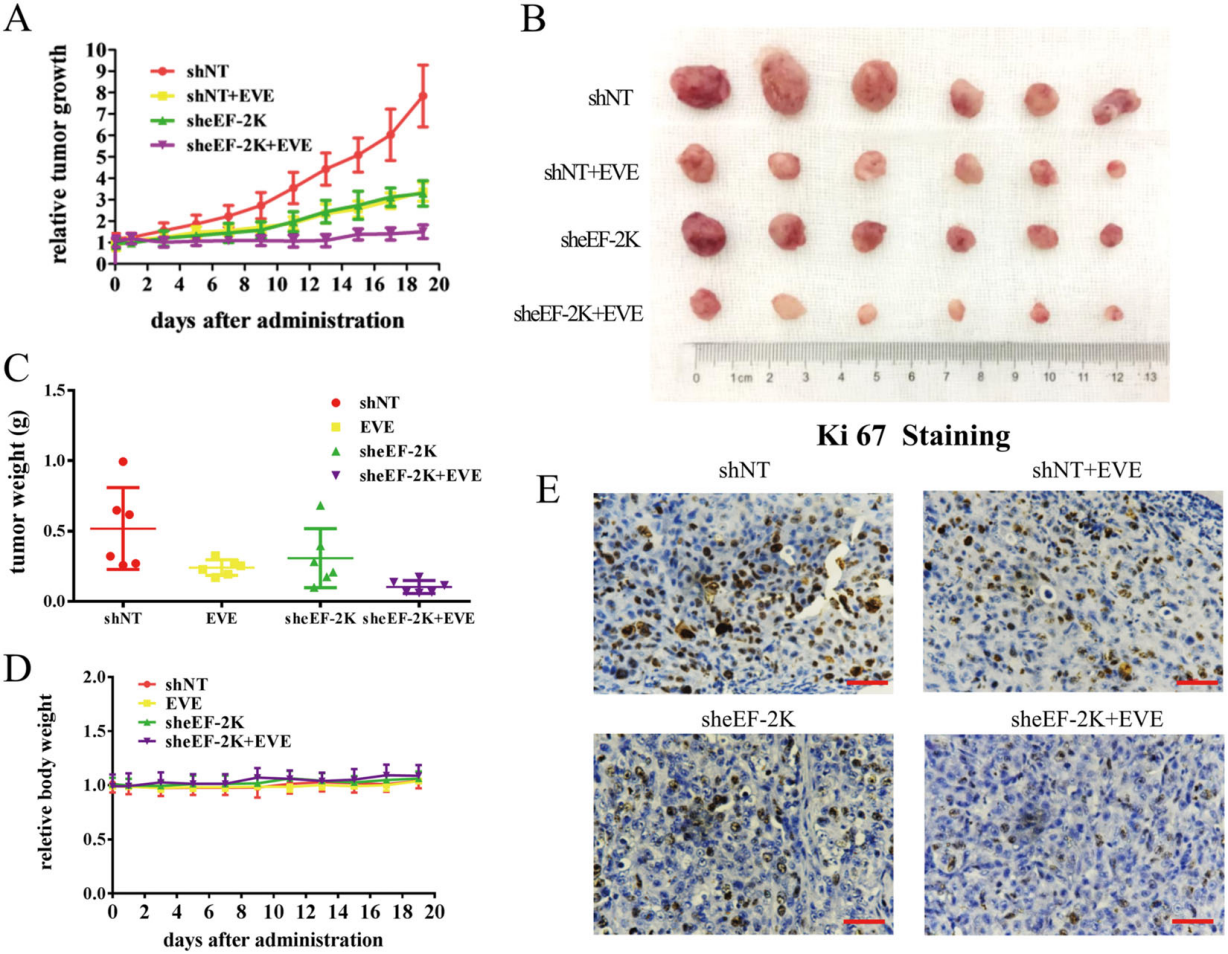
Inhibiting eEF-2K sensitizes breast cancer to everolimus in vivo. **A** Growth curve, **B** dissected tumors, **C** tumor weight, and **D** mouse body weight for the xenograft experiments with MDA-MB-231 cells (shCtrl and sheEF-2K) inoculated subcutaneously into flanks of nude mice treated with 5 mg/kg everlomus. Visible tumors were measured every 2 days. **E** Effect of everolimus treatment on in vivo proliferation was evaluated by IHC staining of ki67 (a marker of proliferative cells). Scale bars: 200 μm.

We further confirmed the synergistic effect of mTOR inhibitor and eEF-2K silencing in vivo. Figure 4A–C shows that cells with knockdown of eEF-2K formed smaller tumors and were more sensitive to everolimus. Meanwhile, there is no significant difference in body weight (Fig. 4D). Ki67 staining demonstrated that everolimus significantly inhibits the growth of eEF-2K knockdown cells. The above results demonstrated that inhibition of eEF-2K significantly reinforces the anticancer efficacy of rapalogs.

### Discovering new eEF-2K inhibitor from FDA-approved drugs

**Fig. 5:**
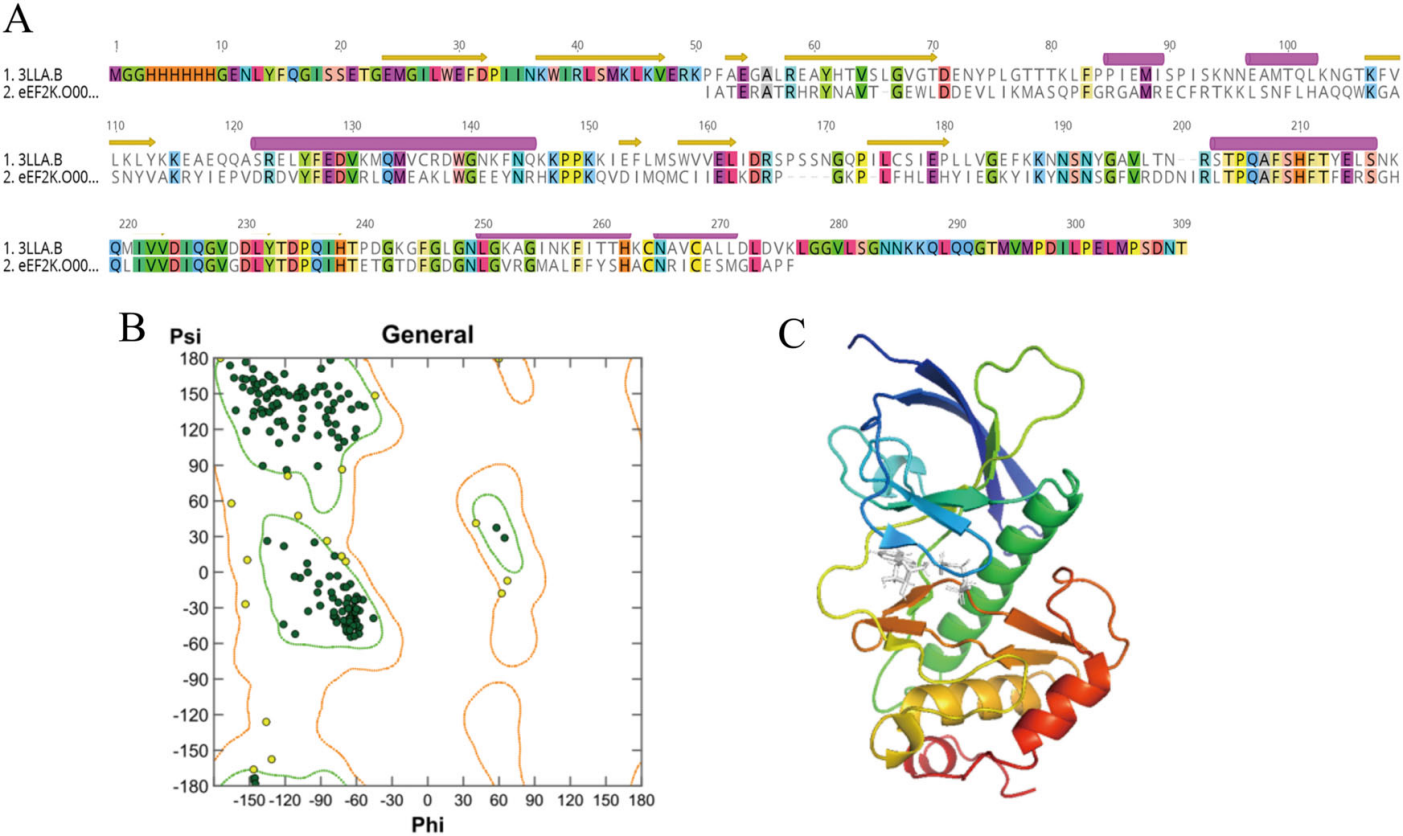
Discovering new eEF-2K inhibitor from FDA-approved drugs. **A** The sequence alignment result between the template protein and eEF-2K. **B** The Ramachandran diagram is used for validating the eEF-2K homology model. **C** The 3D eEF-2K structure from the homology modeling together with its corresponding binding site.

As inhibition of eEF-2K was tightly associated with the enhancement of the anticancer effect of rapalogs, it is high importance to develop eEF-2K inhibitors. Here, docking-based virtual screening was performed to discover novel eEF-2K inhibitors from the FDA-approved Drug Library. First, the 3D crystal structure of eEF-2K was generated for docking studies. Alpha-kinase domain of myosin heavy chain kinase A (MHCKA, PDB ID: 3LKM) was suggested as the template to construct the eEF-2K homology model based on the search results of BLAST, which showed that sequence identity of 42.13% between eEF-2K and MHCKA. The sequence alignment between the template and eEF-2K is shown in Fig. 5A. The Ramachandran diagram was also generated to validate the model, which indicated that more than 90% of the residues were located in the most favored Psi–Phi regions (Fig. 5B). To mimic the active site, AMP was also introduced into the binding pocket of the eEF-2K in the homology modeling. The finally obtained eEF-2K homology model is shown in Fig. 5C.

Due to the poor performance of the present docking protocol, three kinds of docking precision from two docking procedures (Glide and Surflex) were applied to improve docking efficiency. For Glide, Standard Precision (SP) was used to dock hit compounds into the homology model of eEF-2K, and the top 200 compounds based on the docking score were then subjected to Extra Precision (XP) docking, and finally, the top 50 compounds were chosen for induced-fit docking; and the same for Surflex, with a gradual docking of Surflex, Surflex-ck Geom, and Surflex-Dock GeomX step-by-step. Subsequently, the top 30 hit compounds obtained from each protocol were retained for further analysis, and each of the predicted binding patterns was visually inspected, and its interactions within the eEF-2K binding site were also analyzed. According to the reported literature, we concluded that Arg140, Lys170, Ile232, Gly 234, and Tyr236 are key residues for the eEF-2K. Thus, we set a compound selection criterion that the hit compounds should have at least three key residues with eEF-2K. Based on the rule and after removing redundant and non-compliance compounds, seven drugs were suggested as the potential eEF-2K inhibitors and were purchased for further experimental confirmation. We first measured the binding affinity of the seven screened drugs to eEF-2K determined by SPR and found that four drugs (i.e., entecavir hydrate, mitoxantrone, ramelteon, and epalrestat) have a strong binding affinity with eEF-2K (Table 1).

**Table 1:** The binding affinity of the seven screened drugs to eEF-2K determined by SPR.

Compound	Avg KD (M)	Target	Symptom
Entecavir hydrate	2.16 × 10^−6^	Reverse transcriptase	Hepatitis B
Mitoxantrone	9.11 × 10^−6^	Topoisomerase	Anthracenedione antineoplastic agent
Ramelteon	7.71 × 10^−5^	MT	Insomnia
Epalrestat	8.39 × 10^−5^	Others	Diabetic neuropathy
Bezafibrate	5.76 × 10^−4^	PPAR	Hyperlipidemia
Mycophenolic acid	8.44 × 10^−4^	Dehydrogenase	Prevent rejection in organ transplantation
Empagliflozin	9.76 × 10^−4^	SGLT	Type 2 diabetes

### Mitoxantrone blocked the activation of Akt and autophagy induced by mTOR inhibitor by targeting eEF-2K

**Fig. 6:**
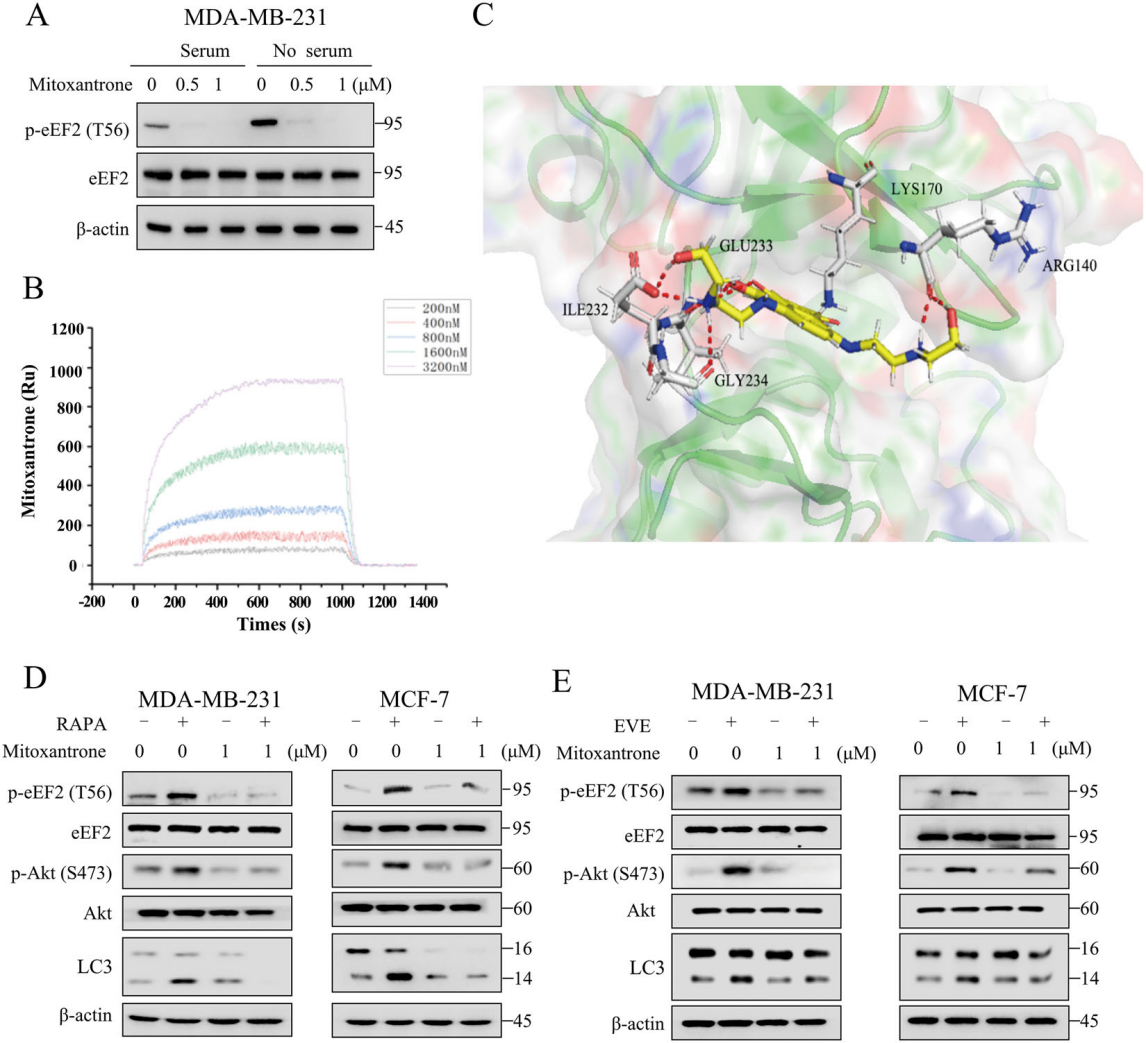
Mitoxantrone blocked the activation of Akt and autophagy induced by mTOR inhibitor by targeting eEF-2K. **A** MDA-MB-231 cells in growth media or starved were treated with indicated concentrations of mitoxantrone for 24 h. Cell lysates were analyzed by western blot for levels of indicated proteins. β-actin was used as a loading control. **B** SPR was performed using purified recombinant eEF-2K with increasing concentrations of mitoxantrone. **C** Schematic illustration of the interaction between eEF-2K and mitoxantrone. **D** MDA-MB-231 or MCF-7 cells were pretreated with 1 μM mitoxantrone for 1 h, followed by treatment with 100 nM rapamycin or everolimus (**E**) for 24 h. Cell lysates were analyzed by western blot for levels of indicated proteins. β-actin was used as a loading control.

We further determined the effects of the drugs on eEF-2K activities by measuring p-eEF2. As shown in Fig. 6A, p-eEF2 was significantly downregulated in the presence of mitoxantrone under standard culturing and nutrient starvation by which eEF-2K can be activated. The kinetic parameters for mitoxantrone and eEF-2K binding are shown in Fig. 6B. According to the specific interaction pattern of mitoxantrone and eEF-2K, we found that there are five hydrogen-bond interactions (Arg140, Lys170, Ile232, Glu 233, and Gly 234) between them (Fig. 6C), and four residues have been reported in the literatures^26–29^, which further demonstrated that mitoxantrone is likely to be a potent inhibitor of eEF-2K.

We next explored whether mitoxantrone could inhibit mTOR inhibitor-induced Akt activation and autophagy. Figure 6D, E show that mitoxantrone simultaneously blocked the activation of Akt and autophagy induced by rapalogs.

### Combination treatment with mTOR inhibitor and mitoxantrone synergistically inhibits the growth of breast cancer cells in vitro and in vivo

**Fig. 7:**
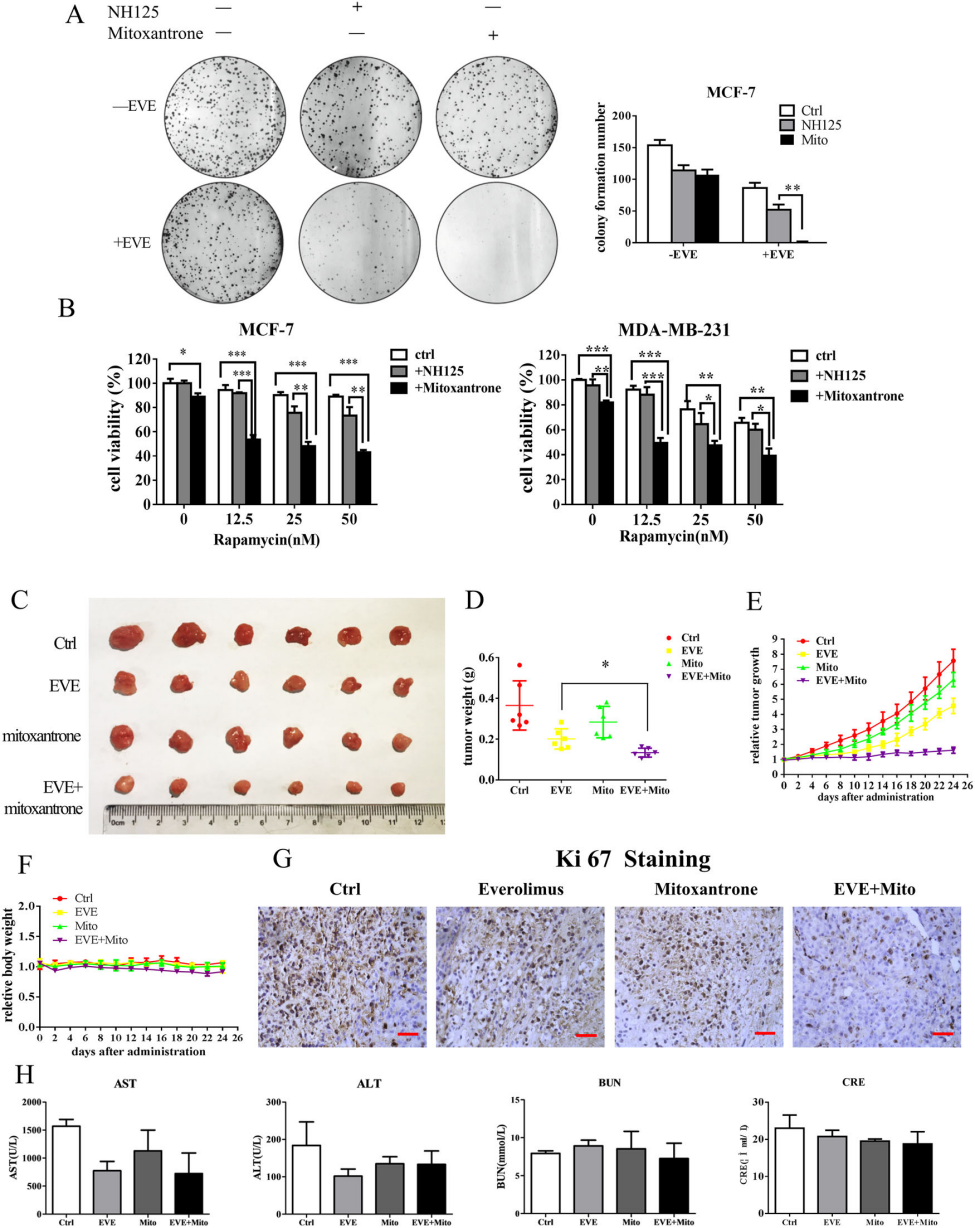
Combination treatment with mTOR inhibitor and mitoxantrone synergistically inhibits the growth of breast cancer cells in vitro and in vivo. **A** MCF-7 cells were treated with 1 μM mitoxantrone or NH125 in the presence or absence of 100 nM everolimus. Colony formation was assessed by crystal violet staining quantification. The results are displayed as means ± SD (*n* = 3). **P < 0.01, *t* test. **B** MCF-7 or MDA-MB-231 cells were pretreated with 1 μM mitoxantrone or NH125 for 1 h, followed by treatment with rapamycin for 72 h. Cell viability was analyzed by CCK8 reagent. The results are displayed as the means ± SD of triplicate measurements from one of three identical experiments; *P < 0.05, **P < 0.01, ***P < 0.001, *t* test. **C** Dissected tumors, **D** tumor weight, **E** growth curve, and **F** mouse body weight for the xenograft experiments with MDA-MB-231 cells inoculated subcutaneously into flanks of nude mice treated with 5 mg/kg everolimus and 0.5 mg/kg mitoxantrone. Visible tumors were measured every 2 days. **G** Effect of everolimus and mitoxantrone treatment on in vivo proliferation was evaluated by IHC staining of ki67 (a marker of proliferative cells). Scale bars: 200 μm. **H** Liver and kidney toxicities were observed after the course of therapy by detecting blood ALT, AST, nitrogen urea, and creatinine.

We next wanted to know whether mitoxantrone can augment mTOR inhibitor-induced cytotoxicity in breast cancer cells by inhibiting eEF-2K. Colony-formation assays showed that the combination of the everolimus and mitoxantrone not only decreased the size of colonies but also significantly reduced the number of colonies (Fig. 7A). Figure 7B shows that co-treatment with mitoxantrone and rapamycin significantly decreased cell viability, as compared with treatment with rapamycin alone, indicating that mitoxantrone sensitizes breast cancer cells to mTOR inhibitors treatment. In addition, we found that NH125, a well-known eEF-2K inhibitor, also further augmented mTOR inhibitor-induced cytotoxicity in breast cancer cells. The combination of mitoxantrone and rapalogs causes a greater growth-inhibitory effect than combined treatment with rapalogs and NH125 (Fig. 7B). We next tested the therapeutic benefits of the combination treatment with mitoxantrone and mTOR inhibitor in vivo. Figure 7C–E, G show that as compared with everolimus alone treatment, co-administration of mitoxantrone and everolimus showed a better therapeutic benefit, as indicated by a significant decrease in the tumor volumes and weights, as well as the proliferative marker Ki67. Combination treatment had no significant changes in body weight (Fig. 7F). Moreover, the dosage of 0.5 mg/kg mitoxantrone and 5 mg/kg everolimus did not induce significant changes in hepatic function or renal function (Fig. 7H). These results collectively suggest that mitoxantrone is a promising eEF-2K inhibitor with a synergistic anti-breast cancer effect in combination with mTOR inhibitors.

## Discussion

**Fig. 8:**
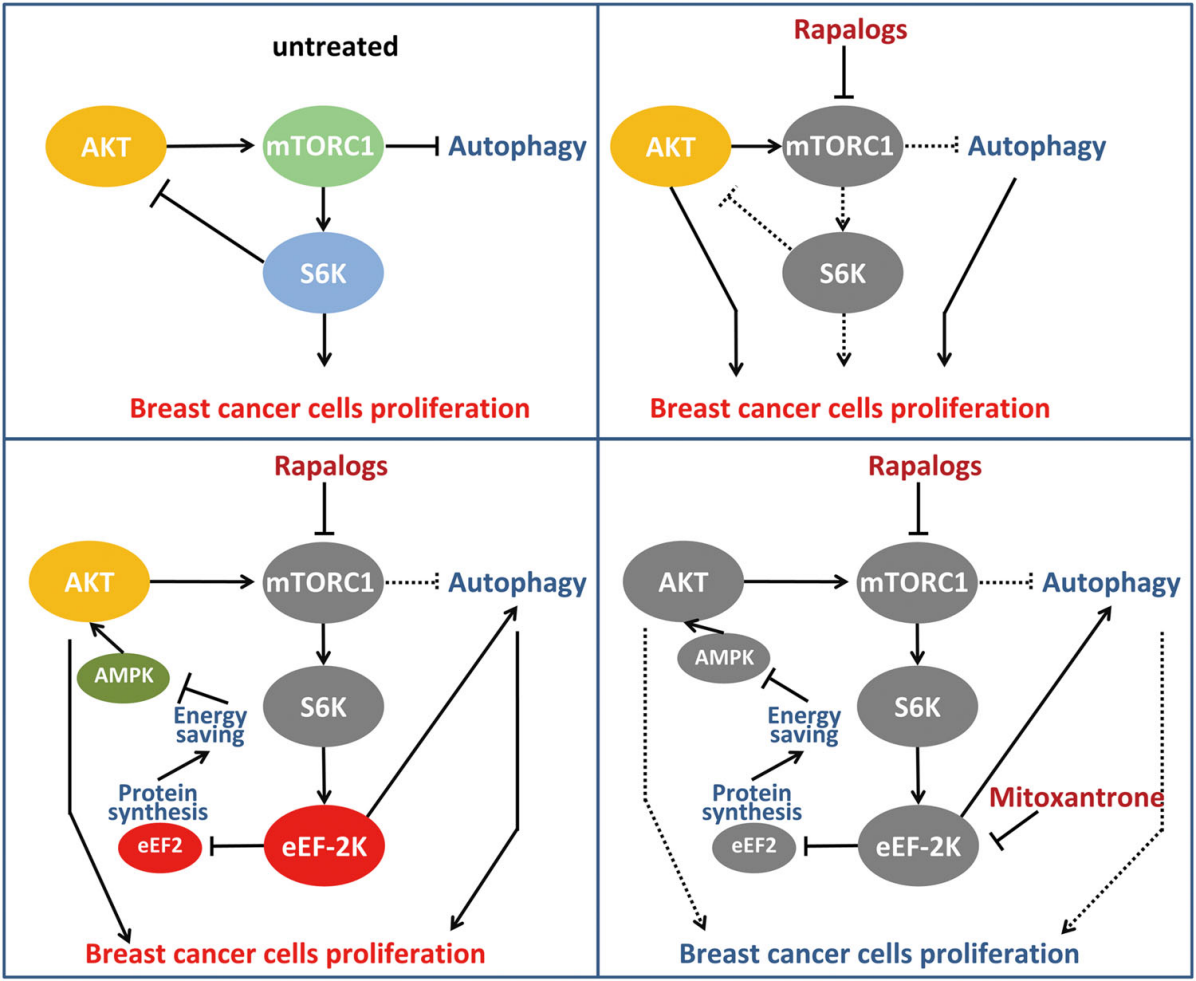
Schematic diagram for eEF-2K-dependent regulation of rapalogs-induced Akt and autophagy activation. The model shows that eEF-2K regulates AMPK activity by controlling the process of peptide elongation, thereby regulating Akt phosphorylation. Mitoxantrone abrogates eEF-2K-mediated Akt activation and autophagy, enhanced mTOR inhibitors anti-breast cancer effect in vivo.

PI3K/Akt/mTORC1 is one of the most frequently mutant pathways in tumor cells, which has given rise to the exploitation of small-molecule inhibitors that target different nodes of the pathway. However, as one of the most promising agents, rapalogs did not exhibit a satisfactory effect in practical application. How to enhance the efficacy of rapalogs remains a challenge in cancer treatment. In this study, we found that targeting eEF-2K could block the two major driven proliferation pathways in response to mTOR inhibitors, thus providing a strategy for conquering the lack of efficiency of rapalogs (Fig. 8).

Negative-feedback relieving leading to overactivation of the oncogene Akt is one of the key elements alleviating rapalogs efficacy. Therefore, the PI3K and mTOR dual-inhibitor PI-103 exhibit a significant inhibitory effect in malignant glioma xenograft^30^. In addition, the mTORC1/mTORC2 dual inhibitors, which not only inhibit mTORC1 but also eliminated mTORC2-dependent Akt activation, thus were developed to solve this problem^31,32^. Herein, we found that eEF-2K is a critical mediator of Akt activity through eEF2-AMPK. It has been well known that AMPK can inhibit Akt through PP2A- or IRS-1-PI3K-mediated signaling pathway^33,34^. AMPK is a major regulator of cellular energy, which can be activated by depletion of ATP. We found that intracellular ATP content was significantly increased when eEF2 was silenced, which is consistent with the promotive role of eEF2 in charge of protein elongation synthesis, which is intense energy dissipation. Consistently, previous study reported that disruption of eEF-2K promotes protein synthesis, which results in ATP consumption and activates AMPK^35^. In addition, the decrease in ATP in eEF-2K knockdown cells was reported by our study that eEF-2K promotes the Warburg effect in cancer cells^20^. Therefore, our results indicate that eEF-2K increases ATP level, thus inhibiting AMPK, resulting in activating Akt.

Autophagy is another side effect in response to rapamycin^36,37^. mTOR inhibitors could activate autophagy by regulating the activity of the ULK protein kinase complex with ATG13, FIP2000, and ATG101 that is required for the formation of autophagosomes^38^. A late phase I/II clinical trial suggests that in patients with renal cell carcinoma, combined 10 mg daily everolimus and 600 mg twice daily hydroxychloroquine was tolerable. Better still, the primary endpoint of >40% 6-month progression-free survival rate was met^15^. It is well documented that eEF-2K is a crucial autophagy regulator demonstrated by our group and others^19,39–41^. Here, we further give the evidence that knockdown eEF-2K abolished rapamycin-induced autophagy which is a pro-survival process, nevertheless the downstream signaling pathway of eEF-2K controlling autophagy remains unclear; further exploration of its mechanism of inducing autophagy will provide new insights for tumor treatment.

Overall, Akt and autophagy activation both lead to mTOR inhibitor resistance and remains an unresolved clinical challenge. In this study, we not only delineated a negative-feedback regulation of Akt by eEF-2K but also demonstrated that eEF-2K serves an important regulatory function in mediating autophagy in response to mTOR inhibitors. Our data showed that disruption of eEF-2K could significantly devastate cancer cell’s capacity to flourish after treatment with rapalogs by regulating cell cycle arrest. The important role of eEF-2K in mediating the resistance of tumor cells to mTOR inhibitors enlightens us that the development of effective inhibitors targeting eEF-2K has a good application prospect and can provide a new strategy for tumor treatment.

Endeavors to develop small-molecule inhibitors toward eEF-2K have been made, however, the outcomes are mixed. NH125 is the widely used eEF-2K inhibitor, but several studies have reported that NH125 is not a specific protein kinase inhibitor as it inhibits other kinases in a similar manner to that of eEF-2K^42^. Moreover, in a number of in vitro assays, NH125 failed to inhibit eEF-2K, instead it led to an increase in the level of eEF2 phosphorylation^43^. A-484954 was a recently reported eEF-2K inhibitor, whereas it exhibits only moderate potency^28^. As effective and specific eEF-2K inhibitor is not available by so far, we wanted to identify new eEF-2K inhibitors by docking-based virtual screening and found that a novel chemotherapeutic agent, mitoxantrone, which exerts its effect by inhibiting topoisomerase II enzyme activity binding to DNA^44^ may be the potential candidate. Mitoxantrone is an analog of doxorubicin; nevertheless it surpasses the efficacy of doxorubicin and has shown promise against several types of malignancies in clinical use^45^. Further experimental evidences indicated that mitoxantrone could bind with eEF-2K and inhibit its activity. We show for the first time that combining mTOR inhibitors and mitoxantrone has synergistic effects against breast cancer cells in vitro and in vivo. These effects are apparently greater than the sum of the inhibitory effects caused by each agent alone, indicating a more than additive or synergistic effect. These results have direct therapeutic implications, suggesting the need for testing this combination in a clinical trial and translate our results into a therapeutic strategy.

In conclusion, our results demonstrated that eEF-2K is involved in mTOR inhibitors-induced activation of Akt and cytoprotective autophagy, and suppression of eEF-2K can significantly reinforce the anticancer efficacy of rapalogs. Thus, these findings provide a mechanistic basis for enhancing mTOR-targeted cancer therapy by combining rapalogs with an eEF-2K inhibitor. Rapamycin combined with mitoxantrone exhibited enhanced inhibitory effects on cancer cells by depressing eEF-2K–driven increase in Akt signaling and cytoprotective autophagy.

## Materials and methods

### Cells lines and culture

MDA-MB-231 and MCF-7 cells were purchased from Cell Bank of Chinese Academy of Sciences, Beijing, China. MCF-7 cells were cultured with DMEM/High glucose (Hyclone) medium supplemented with 10% fetal bovine serum (Gibco) at 37 °C with 5% CO_2_. MDA-MB-231 cells were cultured in L-15 (Hyclone) medium supplemented with 10% fetal bovine serum at 37 °C with 100% air. Cell lines were authenticated using STR profile analysis and used within 3–20 passages of thawing the original stocks.

### Reagents and antibodies

Rapamycin was purchased from Sigma. Everolimus and mitoxantrone were purchased from Selleck. CQ was purchased from Sigma. Antibodies against LC3 (12741#), eEF2 (2332#), phospho-eEF2 (Thr56) (2331), Akt (4691#), phospho-Akt (Thr308) (13038#) phospho-Akt (Ser473) (4060#), p70S6K (2708#), phospho-p70S6K (Thr389) (9234#), phospho-AMPK (Thr172) (2535#), Beclin 1 (4122#), HA (3724#), and tubulin (3873#) were purchased from Cell Signaling Technology. Anti-eEF-2K (ab45168) antibody was purchased from Abcam. Antibodies against Cdk 4 (11026-1-AP), Cdk 6 (14052-1-AP), p21 (10355-1-AP), and β-actin (60008-1-Ig) were purchased from Proteintech. Antibodies against cyclin D1 and AMPK were purchased from Abway. Anti-ki67 was purchased from Maixin (China, Fuzhou).

### Surface plasmon resonance (SPR) assay

The SPR experiments were performed using an Amersham Life Science 110397-04. The compound at 1 mM/L prepared in DMSO was immobilized to a photo-cross-linker SensorCHIPTM. The eEF-2K protein was diluted to a concentration of 200 nM, 400 nM, 800 nM, 1600nM, 3200 nM with PBST (pH = 7.4, 0.5% Tween 20), and PBST serves as the running buffer. eEF-2K protein was injected for 600 s at a flow rate of 0.5 μL/s. Experiments were carried out from low to high concentration; regeneration was carried out by using regenerating solution Glycine HCl (pH = 2.0) at a flow rate 2 μL/s. The dissociation was monitored for 360 s. The experience temperate was 4 °C. Raw data collected on an SPR biosensor were further processed to eliminate any artifacts such as nonspecific binding and discrepancies in buffer composition.

### Plasmid and siRNA transfection

The siRNA targeting eEF-2K and eEF2 were purchased from Gene Pharma and Ribobio, respectively. The siRNA target sequence of eEF-2K was as follows: forward, 5′-GCUCGAACCAGAAUGUCAA-3′. The siRNAs target sequences of eEF2 were as follows: siRNA: 5′-GCUGCGAGCUCCUGUACGA-3′; siRNA 2#: 5′-GCAGUUUGCCGAGAUGUAU-3′; siRNA 3#: 5′-GGACAUCGAUAAAGGCGAG-3′. The eEF-2K shRNA, eEF2 shRNA, and AMPK shRNA were purchased from Genechem. HA-AMPK plasmid was a gift from Xiamen University. Lipofectamine 2000 (Invitrogen) was used for siRNA and plasmid transfection according to the manufacturer’s protocol. In brief, siRNA or 2 μg of plasmid and Lipofectamine 2000 were diluted, respectively, in serum-free DMEM medium at room temperature for 5 min. And then mix the dilution and incubated for 20 min. The mixture was added into the cells and changed into the fresh medium after 4–6-h incubation.

### Western blot analysis

After indicated treatment, cells were lysed by RIPA buffer (Beyotime, P0013B) containing protease inhibitor cocktail and phosphatase inhibitor (Selleck). The cell lysate was centrifuged at 14,000 × *g* at 4 °C for 15 min. The protein concentration of the supernatant was determined by BCA assay (Thermo Fisher Scientific, Darmstadt, Germany). In total, 20 µg of protein samples were subjected to SDS-PAGE and transferred to the PVDF membrane (0.22 µm, Merck Millipore). After blocking with skim milk, the membrane was incubated with the corresponding primary antibodies, followed by HRP-conjugated secondary antibodies. Protein signals were detected by an enhanced chemiluminescence kit.

### Measurement of ATP

Intracellular ATP was measured using ATPlite 1step Luminescence Assay kit (PerkinElmer). Cells with or without silencing eEF2 (5 × 10^3^) were seeded in a 96-well-plate. The lyophilized substrate solution was reconstituted by adding the appropriate-volume buffer to the substrate vial. The medium was discarded in each well and 100 μL of reconstituted reagent was added. The 96-well microplate was shaken for 2 min at 700 rpm using an orbital microplate shaker and then we measured its luminescence.

### Cell viability assay

The cell viability was measured using a CCK8 (Bimake) reagent. Briefly, 5 × 10^3^ cells/well were plated in a 96-well-plate and exposed to various concentrations of drug for indicated durations. After treatment, 10 μL of CCK8 reagent was added into each well and incubated for 2 h. The absorbance was read at 450-nm wavelength.

### Colony-formation assay

In all, 800 cells/well were seeded into a six-well-plate and exposed for indicated treatment for 14 days. The medium was changed every 2 days. After staining with 0.5% crystal violet (Beyotime Biotechnology, Shanghai, China) and washed with PBS, the plates were photographed. The colonies comprising of more than 100 cells were taken into account.

### Edu incorporation assay

Cells were deprived of serum for 48 h to get synchronized and then cultured with a regular medium for 24 h and labeled with Edu reagent for 2 h. Edu incorporation assay (Ribobio) was carried out according to the manufacturer’s instruction.

### Flow-cytometric analysis of cell cycle

After treatment, cells were collected by trypsin and then washed with PBS. The cells were fixed with 70% ethanol for more than 18 h. On the day of analysis, cells were collected and washed twice with cold PBS. Suspend the cells in propidium iodide (PI) staining buffer (BD) and incubated in the dark for more than 15 min. PI-staining cells were analyzed by FACS (BD).

### Mouse xenografts and treatments

All animal experiments were approved by the department of laboratory animals of Central South University. MDA-MB-231 cells (6 × 10^6^/mouse) with or without silencing eEF-2K were injected subcutaneously into the right flank of 6-week-old female nude mice^46^. There were six mice in each group. Once neoplasm size reached 130 mm^3^ (volume = length × width^2^/2), the mice were randomly divided into designed groups and treated for the next 19 days with: (1) vehicle (saline, 100 μL), (2) everolimus (5 mg/kg, oral gavage). For combinational usage of mTOR inhibitor and mitoxantrone, the mice were randomized into four groups and treated with: (1) vehicle (saline, 100 μL), (2) everolimus (5 mg/kg, oral gavage), (3) mitoxantrone (0.5 mg/kg), (4) mitoxantrone (0.5 mg/kg) + everolimus (5 mg/kg). Tumor volume and mice weight were measured every other day.

### Immunohistochemistry (IHC)

Immunohistochemical analysis on the fixed, paraffin-embedded tissue was stained using Ki67 antibody and performed according to the manufacturer’s protocol.

### Docking-based virtual screening

#### Homology modeling of eEF-2K protein

Since the crystal structure of eEF-2K has not been resolved, we modeled the kinase domain of eEF-2K by the homology modeling method in advance. The sequence of the kinase domain of eEF-2K (residues 107–326) was retrieved from the National Center for Biotechnology Institute (NCBI) with UniProtKB accession number (P42527). The protein modeling online server SWISS-MODEL (https://swissmodel.expasy.org/) was employed for the template searching, then the eEF-2K model construction was performed in Molecular Operating Environment (MOE, version 2018). As for its parameter setting, except for the final model parameter of model refinement was set “Fine”, the remaining were with default settings. Subsequently, a variety of protein structure detection tools provided by the online structure detection server SAVES v5.0 (http://servicesn.mbi.ucla.edu/SAVES/) were used to evaluate the quality of the modeled protein, including WHATCHECK, PROCHECK, ERRAT, Verify3D, PROVE, as well as the Ramachandran plot. The detailed model validation information could be found in Supplementary Data.

#### Docking study

Based on the homology model of eEF-2K, molecular docking was carried out to screen potential eEF-2K inhibitors by using Glide and SurFlex. Herein, three docking precision modes of each procedure were employed to improve the efficiency of docking screening, that is, standard precision (SP), extra precision (XP), and induced-fit docking were hierarchically subjected to Glide docking; and a gradual docking of Surflex-Dock, Surflex-Dock Geom, and Surflex-Dock GeomX for Surflex. All settings are default parameters. The screening library, provided by Selleck, was an FDA-approved drug library with 1375 drugs.

### Statistical analysis

All experiments in this study were performed in triplicate, and error bars represent the standard deviation (±SD) of triplicate samples. Statistical analysis was conducted using GraphPad Prism (version 7.0). Comparisons between groups were performed using a two-tailed independent sample Student’s *t* tests analysis. Data were expressed as mean ± SD, *P* < 0.05 was considered a significant difference (*P < 0.05, **P < 0.01, ***P < 0.001).

## Supplementary information

eEF-2K is activated in the presence of rapamycin.

EEF2 promotes Akt dephosphorylation.

Supplementary Figure Legends
