# Supplementary figures and images for: Combined treatment of mitoxantrone sensitizes breast cancer cells to rapalogs through blocking eEF-2K-mediated activation of Akt and autophagy
Source: Cell Death Dis. 2020 Nov 3;11(11):948. doi: 10.1038/s41419-020-03153-x (PMC7642277; doi:10.1038/s41419-020-03153-x)

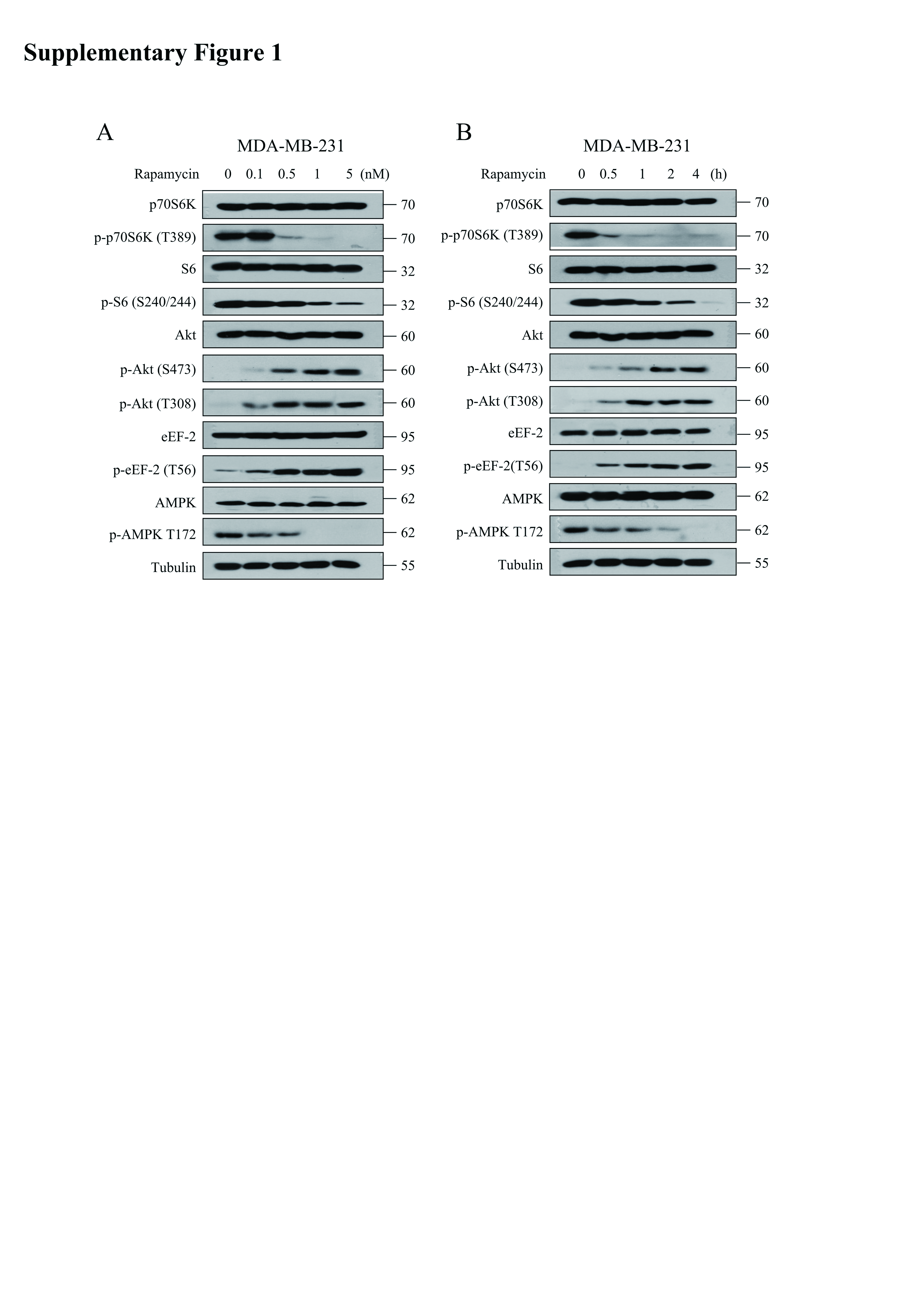

Supplement: Supplementary file 1 — eEF-2K is activated in the presence of rapamycin. [file 41419_2020_3153_MOESM1_ESM.tif]

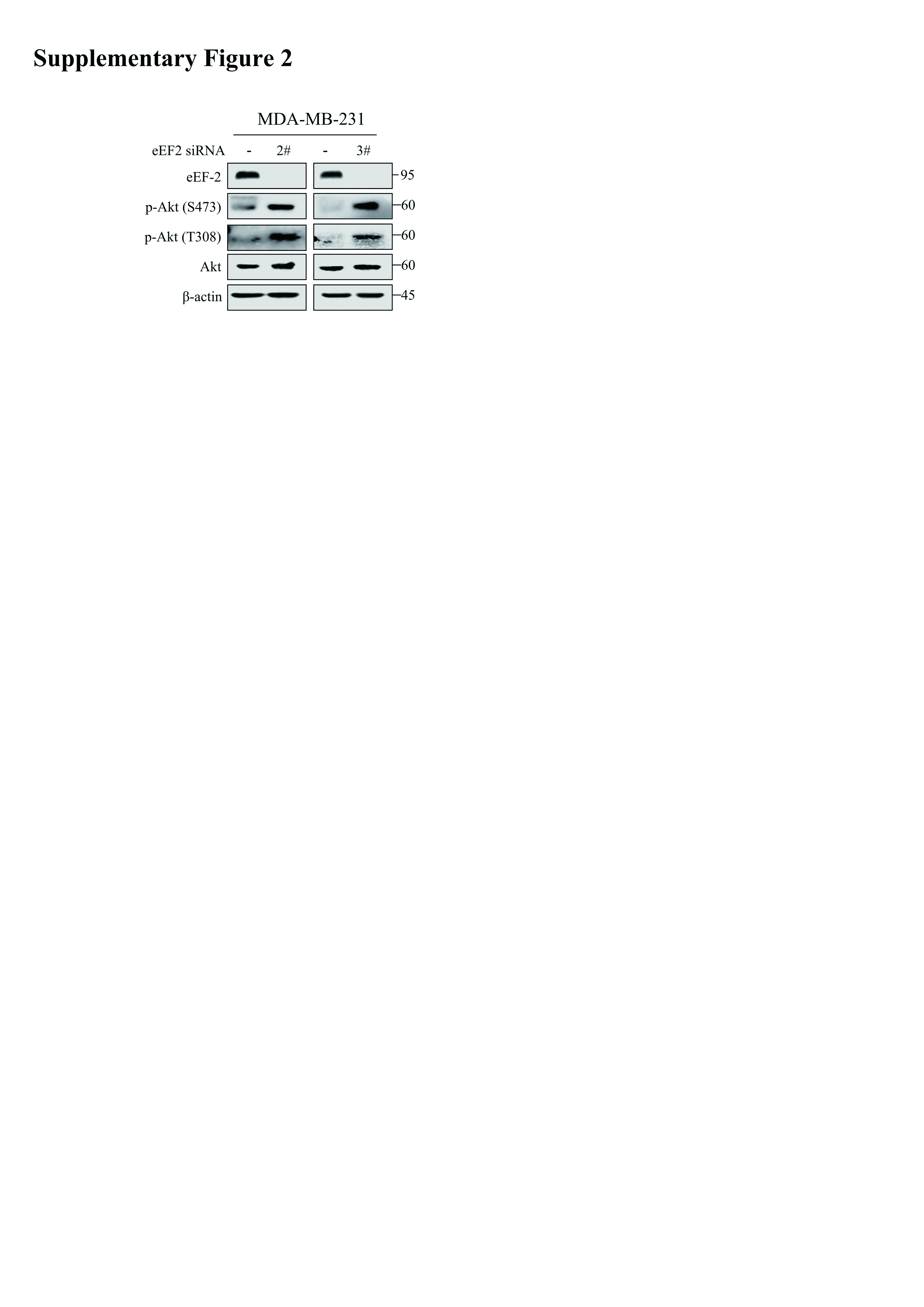

Supplement: Supplementary file 2 — EEF2 promotes Akt dephosphorylation. [file 41419_2020_3153_MOESM2_ESM.tif]
