# Supplementary material for: Combined treatment of mitoxantrone sensitizes breast cancer cells to rapalogs through blocking eEF-2K-mediated activation of Akt and autophagy
Source: Cell Death Dis. 2020 Nov 3;11(11):948. doi: 10.1038/s41419-020-03153-x (PMC7642277; doi:10.1038/s41419-020-03153-x)
Supplement: Supplementary file 3 — Supplementary Figure Legends [file 41419_2020_3153_MOESM3_ESM.docx]

**Table 1**

The binding affinity of the seven screened drugs to eEF2K determined by SPR.

**Supplementary figure 1. eEF-2K is activated in the presence of rapamycin.**

MDA-MB-231 cells were treated with a series of concentrations of rapamycin or with 5nM rapamycin for different durations. Cell lysates were analyzed by Western blot for levels of indicated proteins. Tubulin was used as a loading control..

**Supplementary figure 2. EEF2 promotes Akt dephosphorylation.**

MDA-MB-231 cells were transfected with non-targeting siRNAs or siRNAs targeting eEF2. Cell lysates were analyzed by Western blot for levels of indicated proteins. β-actin was used as a loading control
